# 

**Published:** 1915-09-25

**Authors:** 


### CONTENTS.

| | PAGE |
|---|---|
| **The Limitations of Medical Opinion** ... ... | 533 |
| **The Royal Society of Medicine** ... ... ... | 534 |
| **Hospital and Institutional News** ... ... ... | 535 |
| The Royal Visit to Exeter—The Zeppelin and the Children—Poison in Aeroplane Construction—The End of Cleveland Street Infirmary—German Authorities Uneasy!—D.S.O. for a Naval Surgeon—Economy in Poor-Law Dentistry—A Brotherly Action—War Bonuses for Nurses—Homes of Rest: A Happy Example—"Voluntary Conscription" (?)—Changes in Dietary at Infirmaries—A Notable Absence of Apprehension—The Importance of Oral Hygiene—Poor-Law Guardians and Economy—The Work of the French Red Cross—Mobile Laboratories at the Front—Infant Welfare—York County Hospital—Soldiers Who Refuse Treatment—Refusal to Submit to Operations—The Eastby Sanatorium—Bradford Again—Hospital Gardens—Sickness Benefit in Voluntary Hospitals and Sanatoria—This Week's Drug Market. | |
| **A Medical Causerie** ... ... ... ... ... | 541 |
| **The Insurance Act Drug Tariff** ... ... ... | 542 |
| Drastic Changes Proposed. | |
| **In a General Hospital** ... ... ... ... | 543 |
| II.—Case Notes of an Obscure Disease. History of Case 2. | |
| **National Insurance Act Developments** ... ... | 545 |
| Payments to Hospitals by Insured In-Patients Without Dependants. | |
| **The Work of the R.A.M.C.** ... ... ... ... ... | 547 |
| Another Record of Gallant Deeds. | |
| **Round the World** ... ... ... ... ... ... | 548 |
| Fellow-Workers and the Roll of Honour. | |
| **The Medical Inspection of School Children** ... | 549 |
| Hospitals *v.* School Clinics. | |
| **The War and Infant Welfare** ... ... ... | 550 |
| **The Editor's Letter-Box** ... ... ... ... | 550 |
| R.M.O. and the War Office: Something Wrong Somewhere? | |
| **Hospital Architecture and Construction** ... | 551 |
| Royal Hospital for Sick Children, Glasgow. Adelaide Hospital, South Australia. | |
| **Institutional Needs** ... ... ... ... ... ... | 552 |
| **Latest Installations in Hospital Equipment** ... | 552 |
| **Publishers' Notices** ... ... ... ... ... ... | 52 |
| **The Institutional Worker** ... ... (Suppl.) | 1 |
| Institution Laundries: Saving in Annual Costs. Questions for September. Questions Invited. Enquiries and Answers. Employment and Training. The Sick and In Need. Practical Matters. | |

